# City of London Lying-In Hospital

**Published:** 1905-05-27

**Authors:** 


### CITY OF LONDON LYING-IN HOSPITAL.

The first festival dinner in aid of the City of London Lying in Hospital, which has been held for over 50 years, took place at De Keyser's Royal Hotel on Tuesday, May 23rd, the Lord Mayor in the chair. The purpose of the dinner was to assist in raising funds for the rebuilding of the old portion of the hospital, which has been rendered necessary by the very dilapidated condition into which the premises have fallen after having been in use for considerably over 100 years. About £35,000 is required for this purpose, towards which £2,625 was collected at the dinner.

The Lord Mayor in proposing "Prosperity to the City of London Lying-in Hospital," said that the appeal they were making was not the outcome of any rebuilding craze, was not the result of ambition on the part of those responsible for the management of the hospital, but it was an absolute necessity caused by the circumstance that within the last few years there had been two railways made under the City Road, the effect of which had been to cause a subsidence of soil that had endangered the hospital buildings. In fact, before the Committee of Management had come to the conclusion to rebuild, the hospital had been condemned by the local authorities as insecure. Every care had been taken to provide for the erection of suitable buildings, but, notwithstanding the exercise of all possible economy, the new hospital could not be completed for less than £35,000. The hospital was the only lying-in hospital so situated as to be able to minister to the needs of poor districts like Hoxton, Bethnal Green, Shoreditch, St. Luke's, and Islington. He felt that no other metropolitan institution was doing the same practical, necessary, and beneficial work, nor one which had stronger claims upon the charitable than that of the hospital for which it was his privilege to plead that even ing. (Applause.)

Replying to the toast of "The Chairman and Medical and Surgical Staff," Dr. Clement Godson mentioned in support of the Lord Mayor's statement that this question of rebuilding was no new thing, and that as far back as 1882 a special Court of Governors proposed that the hospital should be pulled down and rebuilt.

